# Reevaluating drivers of endometriosis burden: a call for deeper contextualization

**DOI:** 10.1097/JS9.0000000000003096

**Published:** 2025-07-18

**Authors:** Haoyuan Li, Haiying Fan, Yiwen Yang

**Affiliations:** a Shanghai Fengxian District Traditional Chinese Medicine Hospital, Shanghai, China;; b Fengpu Street Community Health Service Center, Fengxian District, Shanghai, China

*Dear Editor,*

We read with great interest the recent article by Xu and colleagues entitled “Global, regional, and national burden of endometriosis among women of childbearing age from 1990 to 2021: a cross-sectional analysis from the 2021 global burden of disease study,” published in the *International Journal of Surgery*[1]. This comprehensive study provides valuable insights into the temporal and spatial patterns of endometriosis prevalence and burden across diverse global regions, with forward-looking projections extending to 2040. While commending the breadth and depth of this research, we would like to raise several points for clarification and further consideration. This study is compliant with the TITAN Guidelines 2025—governing declaration and use of AI[2].

First, the authors attribute part of the observed global declines, particularly in low-income regions, to public health efforts such as Australia’s National Action Plan for Endometriosis (NAPE). However, this extrapolation may be misleading. NAPE is a domestic initiative targeting the Australian population, and its applicability to global trends is questionable. In fact, since the implementation of NAPE, national hospitalization data from Australia reveal a marked increase in endometriosis-related admissions, particularly among younger women, alongside a notable decrease in the median age at diagnosis[3]. These trends likely reflect heightened awareness, enhanced diagnostic capacity, and broader access to care, rather than a genuine reduction in disease incidence. Therefore, caution should be exercised when extrapolating such national interventions to global trends.

Second, the study’s broad explanations for regional disparities, such as socioeconomic factors, ethnicity, and education, would benefit from further exploration. Cultural normalization of menstrual pain often leads to diagnostic delays, as symptoms are trivialized by both patients and health providers[4]. Moreover, structural inequities within healthcare systems exacerbate these delays for marginalized populations. For example, rural, Black, and Hispanic women experience longer diagnostic intervals and reduced access to specialist care compared to White women[5]. These sociocultural and systemic barriers, coupled with environmental exposures and genetic predispositions[6], contribute to the observed epidemiological differences.

To summarize, while the study by Xu *et al.* contributes significantly to the global understanding of endometriosis, nuanced interpretation of regional trends and causality is essential to inform equitable and effective health strategies.

## Data Availability

All data are included in this article.
